# 205. Evaluating the Time to Oral Step-Down Therapy for Gram-Negative Bloodstream Infections Associated with a Genitourinary Source

**DOI:** 10.1093/ofid/ofab466.407

**Published:** 2021-12-04

**Authors:** Nicole Mulvey, Thien-Ly Doan, Lemar Nadi

**Affiliations:** 1 Long Island Jewish Medical Center, New Hyde Park, New York; 2 Northwell Health, Hicksville, New York

## Abstract

**Background:**

The transition to oral antibiotics in gram-negative bloodstream infections (BSI) can decrease length of stay, avoid central line access, and improve patient convenience. Some studies suggest that the bioavailability of the oral agent selected can impact outcomes. The purpose of this study was to determine when the most appropriate time to oral step-down is, and assess if the bioavailability of the agent selected impacts outcomes.

**Methods:**

This retrospective observational chart review evaluated adult patients admitted to Long Island Jewish Medical Center during the study period of January 2019 – December 2019 with a gram-negative BSI from a genitourinary source. The primary objective was to assess if the time to oral step-down therapy impacts clinical success. Secondary objectives included assessment of if continued IV therapy or oral step-down impacts outcome measures including clinical failure, length of stay, and duration of therapy, and to compare high versus low bioavailability agents on treatment outcomes.

**Results:**

A total of 130 patients were included, with 88 patients in the oral step-down group and 42 patients in the IV therapy only group. Clinical failure occurred in 10 patients in the oral step-down group, with 2 de-escalated in the 1-3 day range and 8 de-escalated in the 4-6 day range (p=0.29). There was no difference in clinical failure when the oral step-down group was compared to the IV therapy group (11 vs. 17%; p=0.41). The length of stay was significantly shorter in the oral step-down group (p< 0.0001), while the duration of therapy was shorter in the IV therapy group (p=0.0015). When comparing high and low bioavailability agents, there was no difference in the rate of treatment failure (p=0.74), length of stay (p=0.08), or duration of therapy (p=0.02).

**Conclusion:**

There was no significant difference in outcomes if patients were de-escalated to oral therapy early versus late in their treatment course. Step-down to oral antibiotics led to decreased length of stay, and the bioavailability of the oral agent selected did not impact outcomes. This study demonstrates the safety and efficacy of prompt oral step-down for gram-negative bacteremia secondary to a genitourinary source which can have positive impacts on patient care.

**Disclosures:**

**All Authors**: No reported disclosures

